# A Novel 3D Magnetic Resonance Imaging Registration Framework Based on the Swin-Transformer UNet+ Model with 3D Dynamic Snake Convolution Scheme

**DOI:** 10.3390/jimaging11020054

**Published:** 2025-02-11

**Authors:** Yaolong Han, Lei Wang, Zizhen Huang, Yukun Zhang, Xiao Zheng

**Affiliations:** School of Computer Science and Technology, Shandong University of Technology, Zibo 255049, China; 18753361907@163.com (Y.H.); 13145385178@163.com (Z.H.); zyk6652@126.com (Y.Z.); xiao_zheng0910@163.com (X.Z.)

**Keywords:** magnetic resonance imaging, image registration, swin transformer, UNet, 3D dynamic snake convolution, weakly supervised

## Abstract

Transformer-based image registration methods have achieved notable success, but they still face challenges, such as difficulties in representing both global and local features, the inability of standard convolution operations to focus on key regions, and inefficiencies in restoring global context using the decoder. To address these issues, we extended the Swin-UNet architecture and incorporated dynamic snake convolution (DSConv) into the model, expanding it into three dimensions. This improvement enables the model to better capture spatial information at different scales, enhancing its adaptability to complex anatomical structures and their intricate components. Additionally, multi-scale dense skip connections were introduced to mitigate the spatial information loss caused by downsampling, enhancing the model’s ability to capture both global and local features. We also introduced a novel optimization-based weakly supervised strategy, which iteratively refines the deformation field generated during registration, enabling the model to produce more accurate registered images. Building on these innovations, we proposed OSS DSC-STUNet+ (Swin-UNet+ with 3D dynamic snake convolution). Experimental results on the IXI, OASIS, and LPBA40 brain MRI datasets demonstrated up to a 16.3% improvement in Dice coefficient compared to five classical methods. The model exhibits outstanding performance in terms of registration accuracy, efficiency, and feature preservation.

## 1. Introduction

In recent years, with the rapid advancement of medical technology, particularly in medical imaging, magnetic resonance imaging (MRI) has become increasingly essential in clinical diagnosis, especially for cerebrovascular diseases and intracranial tumors [1]. However, due to factors such as imaging time, angle, parameters, and equipment, MRI images acquired at different time points or angles exhibit spatial differences, which can complicate diagnosis. To assist doctors in handling complex diagnostic situations, a series of advanced technologies are required, such as the latest attention-mechanism-based LSTM-AE method for predicting spinal surgical respiratory motion in spinal surgery [2], the widespread application of laser technology in cancer treatment [3], and the use of ultrasound-guided microscopy in evaluating type 2 diabetes [4]. Furthermore, image registration techniques are essential for helping doctors accurately compare and analyze lesions across different time sequences and modalities. Medical image registration is a computer vision and image processing technique aimed at aligning two or more images obtained under different conditions (e.g., time, sensor, angle) into a common coordinate system for comparison or fusion [5]. Early image registration methods included manual registration, rigid transformation-based methods, and deep-learning-based approaches [6,7]. As registration technology has evolved, deep-learning-based methods have gradually replaced other approaches and become the mainstream technique, with widespread applications.

The principle of deep-learning-based registration methods is to train neural network models to learn the complex deformation relationships between images, enabling one image to warp and align with another [8]. The first deep-learning-based image registration model, VoxelMorph, was proposed by Balakrishnan et al. This model employs convolutional neural networks (CNNs) with spatial transformation layers to reconstruct registration results. It learns complex nonlinear transformations from large datasets, achieving a higher registration accuracy compared to traditional methods [9]. To address the challenge of CNN-based registration methods in handling long-range dependencies between moving and fixed images [10], researchers have developed Transformer-based approaches. For instance, Chen et al. introduced the ViT-V-Net model [11], which incorporates the self-attention mechanism of vision transformer (ViT) to globally model pixel relationships. This enables the model to effectively capture long-range dependencies between moving and fixed images, demonstrating excellent performance in medical image registration tasks involving significant deformations. Subsequently, to reduce the high computational demands of Transformer-based registration methods, researchers proposed swin-transformer-based models. Jun et al. introduced TransMorph [12], which combined a swin transformer with convolutional modules to efficiently capture long-range dependencies and global context. By replacing global attention with local attention, a swin transformer significantly reduces the computational and memory complexity [13]. However, these models face limitations in representing fine local features and reconstructing global contextual information. Fragmentation issues lead to an incoherent context and a lack of comprehensive global representation. To enhance detail processing, researchers have proposed models with dual consistency constraints and prior-knowledge-based loss functions. For example, Huang et al. introduced an unsupervised coarse-to-fine multi-contrast MR image registration model [14], which improves registration accuracy and detail representation by addressing pixel folding issues. While effective in handling various MR contrasts, these models still require optimization to manage complex organ deformations and fully represent global and local features.

The aforementioned methods are unsupervised registration approaches, which, while reducing the reliance on annotated data, generally exhibit a lower accuracy compared to supervised methods. Due to the high cost of annotating medical MRI datasets, achieving a performance comparable to supervised methods using unsupervised techniques has become a key research focus. To further enhance the registration performance and reduce the reliance on labeled data, Alexander Bigalke et al. proposed a self-supervised registration method [15,16]. In the initial stage, the model is trained with a small amount of data, and an optimization process guides self-training to ensure that the generated pseudo-labels effectively improve the model’s accuracy. These pseudo-labels, predicted by the model, are then used to further optimize the training process. However, the quality of the pseudo-labels directly impacts the training outcome. If the optimization process does not sufficiently improve the accuracy of the pseudo-labels, the model may converge to suboptimal solutions or even amplify errors. Improving prediction accuracy during the registration stage is crucial for enhancing the effectiveness of weakly supervised methods.

In summary, this paper proposes a novel deformable medical image registration framework, referred to as the optimized weakly supervised swin transformer-UNet combined with 3D dynamic snake convolution (OSS DSC-STUNet+). This framework is built upon the swin transformer algorithm and the UNet architecture, with extensions to the Swin-UNet model. The framework consists of two stages: the registration stage, and the optimization stage. In the registration stage, the proposed 3D DSC-STUNet+ model generates deformation fields and registered images. Inspired by the concepts of weak supervision and optimization, the optimization stage integrates these principles into the OSS DSC-STUNet+ model to iteratively refine the generated deformation field. Together, these two components form the foundational architecture of our image registration method.

The contributions are recapped as follows:

1. To enhance the accuracy of the Swin-UNet model in handling MRI images with rich details, we extend the latest dynamic snake convolution to 3D space and integrate it into the Swin-UNet architecture, improving the model’s ability to capture fine features.
2. The Swin-UNet structure is optimized by increasing the network depth and incorporating multi-scale dense connections, which enhance the model’s ability to extract both global and local features from moving and fixed images, while maintaining the overall structural integrity of the image.
3. A weakly supervised optimization strategy is incorporated into the model, dynamically generating pseudo-ground truth deformation fields during training to supervise the model. By improving the accuracy of the registration model, the weakly supervised optimization strategy further enhances the overall registration accuracy.

## 2. Related Work

### 2.1. Deformable Image Registration

**Figure 1:**
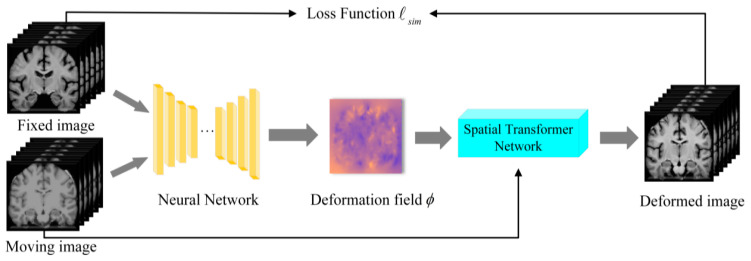
Deep-learning-based image registration architecture.

Deformable image registration (DIR) refers to the process of aligning two or more images by allowing for local transformations, to account for differences in shape, size, or anatomy. Unlike rigid registration, which only allows for global transformations like translation and rotation, deformable registration permits more complex deformations, enabling more accurate alignment of images with structural variability. This flexibility makes DIR valuable in medical imaging applications. The mathematical model for deformable image registration can be expressed as [17]

(1) $$\hat{\phi }=\arg\underset{\phi }{\min}l(I_{m}\circ \phi ,I_{f})+\lambda R(\phi )$$

where *ϕ* represents the deformation field that warps the moving image (*I_m_* > *ϕ*), and *λ* is a regularization hyperparameter that controls the trade-off between image similarity and the smoothness of the deformation field. However, these traditional methods are computationally expensive and have limited generalization capabilities. Subsequently, deep-learning-based registration methods have been introduced, where the principle is to train neural network models to learn complex deformation relationships between images, allowing one image to be deformed and aligned with another. The basic architecture of deep-learning-based image registration is shown in Figure 1.

### 2.2. 3D Swin Transformer Block

The swin transformer module comprises the following components: a LayerNorm (LN) layer, a multi-head self-attention (MSA) mechanism, residual connections, and a two-layer MLP with GELU activation [18]. The 3D swin transformer block was designed based on a shifted window approach. In this design, a window-based multi-head self-attention (W-MSA) mechanism is utilized in the first block, while a shifted window-based multi-head self-attention (SW-MSA) mechanism is applied in the second block. This dual-module design enables the swin transformer to effectively capture both global and local feature information. The sequential swin transformer blocks can be represented as

(2) $$\begin{array}{l} {\hat{z}}_{l}=W-MSA(LN(z_{l-1}))+z_{l-1}\\ z_{l}=MLP(LN(\hat{z}))+{\hat{z}}_{l}\\ {\hat{z}}_{l+1}=SW-MSA(LN(z_{l}))+z_{l}\\ z_{l+1}=MLP(LN({\hat{z}}_{l+1}))+{\hat{z}}_{l+1} \end{array}$$

where ${\hat{z}}_{l}$ and *Z_l_* correspond to the output features of the W-MSA3D (or SW-MSA3D) and MLP modules, respectively. Here, W-MSA3D and SW-MSA3D denote the window-based and shifted window-based multi-head self-attention mechanisms in 3D, while MLP represents the multilayer perceptron module.

The overall architecture of the model in this paper is primarily based on a UNet framework constructed using a swin transformer, which forms the encoder and decoder components of the model.

## 3. Methodology

### 3.1. The Proposed Registration Network

#### 3.1.1. Overall Structure

We propose a magnetic resonance imaging registration model based on Swin-Unet, incorporating optimization and supervised learning concepts into the training process. In the subsequent sections, *I_m_* and *I_f_* represent the moving and fixed images, respectively, while *ϕ* and *ϕ_opt_* denote the deformation field generated by the registration model and the deformation field optimized by the optimizer, respectively.

**Figure 2:**
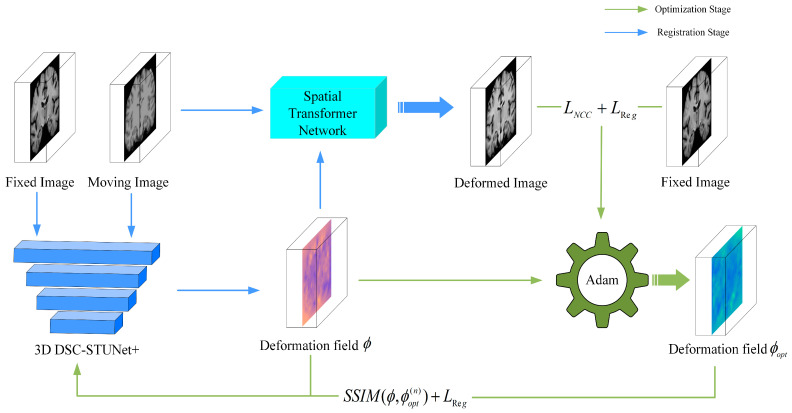
Structure of OSS DSC-STUNet+ network model.

As shown in Figure 2, our model consists of two main components. The registration part utilizes our proposed 3D DSC-STUNet+ model to perform registration, outputting the deformation field *ϕ* and the deformed image. The optimization part compares the fixed image with the deformed image using a loss function, and the loss function’s output, along with the deformation field *ϕ* is used to supervise the model by generating a pseudo-ground truth deformation field *ϕ_opt_* through the Adam optimizer.

#### 3.1.2. The Structure of the 3D DSC-STUNet+ Network Model

**Figure 3:**
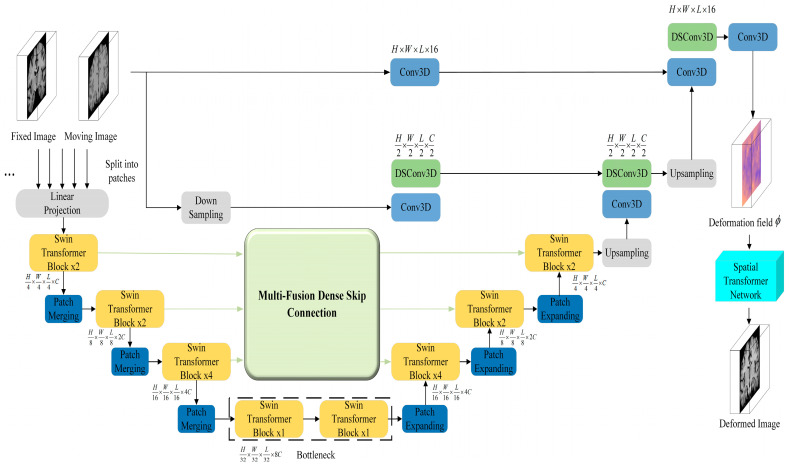
Structure of 3D DSC-STUNet+ network model.

As shown in Figure 3, the structure of the 3D DSC-STUNet+ network model primarily consists of four consecutive swin transformer downsampling modules, four consecutive swin transformer upsampling modules, two skip connections and a multi-fusion dense skip connection between the swin transformer modules, a basic UNet network composed of two consecutive upsampling convolution layers, and subsequent upsampling modules. The 3D DSC-STUNet model was built upon the encoder–decoder architecture of UNet. Unlike the fully symmetric structure of a conventional UNet, this model uses a Swin-UNet, which combines a swin transformer with UNet, as the backbone network for extracting key features. In the final upsampling stage of a typical Swin-UNet, the image is restored using a Patch Expand 4x layer. In our model, this Patch Expand 4x layer is replaced with two consecutive convolutional blocks for further upsampling. The second layer of these two consecutive convolutional blocks is replaced by a dynamic snake convolution. Additionally, spatial and channel attention modules (Spatial and Channel Squeeze and Excitation, scSE) [19] are introduced after both convolutional blocks to better capture spatial and channel correlations in the feature maps. This enables the network to automatically focus on important features, while suppressing less relevant ones, thus improving the model’s performance and generalization capabilities.

The basic unit of Swin-UNet is the swin transformer module [18,20]. In the encoder phase, the network first divides the input moving and fixed images into non-overlapping 3D patches of size 2 × 4 × 4 × 4. These patches are then flattened and represented as tokens, which are projected into arbitrary dimensions (denoted as C) using a linear embedding layer. The transformed patch tokens are passed through multiple swin transformer modules and patch merging layers to generate hierarchical feature representations. the swin transformer module extracts high-resolution features from magnetic resonance images (MRI), producing hierarchical feature representations. The decoder is composed of swin transformer blocks and patch expanding layers. The patch expanding operation gradually reduces the number of channels, while increasing the spatial resolution of the feature maps. In the decoder phase, hierarchical features are fused with corresponding upsampled features, effectively transferring the low-level detail information lost during downsampling to the corresponding layers of the decoder. This helps recover fine details in the image and achieves more accurate localization.

After the upsampling stage of the swin transformer, the model uses two consecutive convolutional layers to restore the (*H*/4) × (*W*/4) × (*L*/4) × *C* sized feature map processed by the swin transformer block into a *H* × *W* × *L* × 16 sized feature map. These two consecutive convolutional layers consist of a Conv3D layer and a DSConv3D layer. The Conv3D layer includes a 3 × 3 × 3 convolution with a kernel size of 3, stride of 1, and padding of 1, followed by a batch normalization layer and a ReLU activation function. After the Conv3D layer, a 3D dynamic snake convolution (DSConv3D) is applied to enhance the local details and features of the feature map (DSConv3D will be further discussed in the next section).

The subsequent upsampling phase continues to employ a skip connection strategy, where the feature maps of dimensions (H/2) × (W/2) × (L/2) × (C/2), generated from the downsampling followed by convolution of the moving image, and the feature maps of dimension H × W × L × 16, generated from direct convolution, are concatenated with the feature maps produced by the upsampling convolution block. This process enhances the spatial details of the feature maps. After the upsampling phase, the deformation field *ϕ* is generated. Finally, the spatial transformer network (STN) [21] applies nonlinear transformation to the moving image I_m_, resulting in the final registered image.

**Figure 4:**
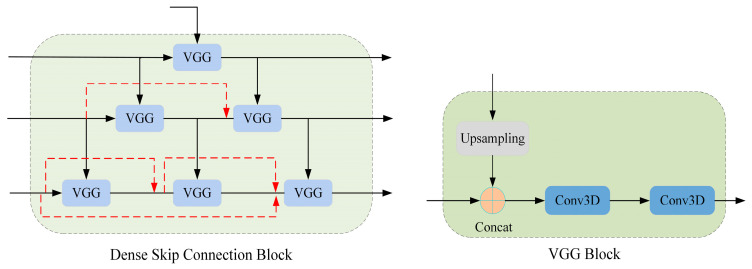
The multi-fusion dense skip connection block.

Inspired by SCUNet++ [22], we added multi-fusion dense skip connections to our registration model. These skip connections combine nested and multi-fusion designs with both long and short connections, as shown in Figure 4. They include multiple VGG module layers, which downsample the input and concatenate it with the current layer’s input. Two Conv3D modules then process the features of the skip connections. This design captures multi-level features across different receptive fields, reducing the detail loss in feature extraction caused by the transformer-based Swin-UNet structure.

In the registration phase of the model, the 3D DSC-STUNet+ registration model predicts a deformation field *ϕ* for each pair of input moving images *I_m_* and fixed images *I_f_*, i.e.,

(3) $$F_{\theta }(I_{f},I_{m})=\phi$$

where *θ* represents the parameters of the registration network. The deformation field generated by our model during the registration phase serves as the initial parameters for further optimization in the optimization phase.

### 3.2. 3D Dynamic Snake Convolution

To enhance the local features of input images, Conv3D was introduced in the proposed model. However, conventional convolution is not sufficiently sensitive to elongated local structures in medical images. For this reason, dynamic snake convolution (DSC) [23] was incorporated into our model. Therefore, we extended this convolution to three-dimensional space and optimized it to better adapt to our architecture. Originally designed to improve the segmentation accuracy and efficiency of complex and variable fine features, such as blood vessels and roads, dynamic snake convolution focuses adaptively on slender and intricate local structures. This enables the precise extraction of tubular and complex patterns. In this study, MRI images of the human brain, especially in the cerebellum, exhibited highly intricate local features, which significantly increased the difficulty of registration. By integrating 3D dynamic snake convolution, the ability of our model to extract these local fine features is effectively enhanced.

**Figure 5:**
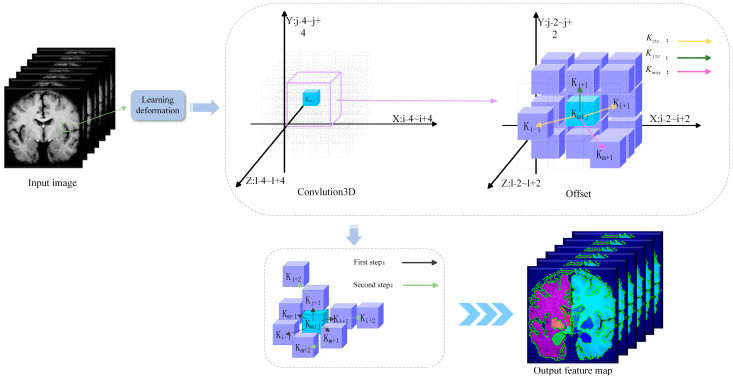
Schematic diagram of 3D dynamic snake convolution.

As shown in Figure 5, dynamic snake convolution is based on the ideas of dynamic convolution and deformable kernel convolution [24,25]. Given the standard 3D convolution coordinates K, with the convolution center at *K_i_* = (*x_i_*, *y_i_*, *z_i_*), a 3 × 3 × 3 convolution kernel can be expressed as

(4) $$\begin{array}{r} K=\{(x-1,y-1,z-1),(x-1,y-1,z),\ldots ,\\ \ldots ,(x,y+1,z+1),(x+1,y+1,z+1)\} \end{array}$$

To enhance the flexibility of the convolution kernel and enable it to capture the complex geometric features of the target, dynamic snake convolution introduces a deformable offset, denoted as Δ. However, when dealing with elongated structures, allowing the model complete freedom to learn the deformable offsets can cause the receptive field to deviate from the target. This issue is addressed by employing an iterative strategy, as shown in Figure 5. This strategy incrementally selects the next position of the target for observation, ensuring continuity in feature focus and preventing the receptive field from over-expanding due to large deformable offsets.

In dynamic snake convolution, the standard convolution kernel is linearized along the *x*, *y*, and *z* axes. Taking the *x*-axis as an example for a convolution kernel of size 3 × 3 × 3, the specific positions of each grid in the kernel *K* are represented as

(5) $$K_{i\pm c}=(x_{i\pm c},y_{i\pm c},z_{i\pm c})$$

The transformations along the *x*, *y*, and *z* axes are represented as follows:

(6) $$\begin{array}{c} K_{i+c}=(x_{i+c},y_{i+c},z_{i+c})\\ \;\;\;\;=(x_{i}+c,y_{i}+\sum_{i}^{i+c}\Delta y,z_{i}+\sum_{i}^{i+c}\Delta z),\\ K_{i-c}=(x_{i-c},y_{i-c},z_{i-c})\\ \;\;\;\;=(x_{i}-c,y_{i}+\sum_{i-c}^{i}\Delta y,z_{i}+\sum_{i-c}^{i}\Delta z)\\ K_{j+c}=(x_{j+c},y_{j+c},z_{j+c})\\ \;\;\;\;=(x_{j}+\sum_{j}^{j+c}\Delta x,y_{j}+c,z_{j}+\sum_{j}^{j+c}\Delta z),\\ K_{j-c}=(x_{j-c},y_{j-c},z_{j-c})\\ \;\;\;\;=(x_{j}+\sum_{j-c}^{j}\Delta x,y_{j}-c,z_{j}+\sum_{j-c}^{j}\Delta z)\\ K_{m+c}=(x_{m+c},y_{m+c},z_{m+c})\\ \;\;\;\;=(x_{m}+\sum_{m}^{m+c}\Delta z,y_{m}+\sum_{m}^{m+c}\Delta y,z_{m}+c),\\ K_{m-c}=(x_{m-c},y_{m-c},z_{m-c})\\ \;\;\;\;=(x_{m}-\sum_{m-c}^{m}\Delta z,y_{m}+\sum_{m-c}^{m}\Delta y,z_{m}-c) \end{array}$$

Since the offset Δ is typically a fractional value, while the coordinates are usually in integer form, trilinear interpolation is applied. This can be expressed as

(7) $$K=\sum_{K^{\prime }}B(K^{\prime },K)\cdot K^{\prime }$$

where *K* represents the fractional positions in Equation (7), *K*′ lists all the integer spatial positions, B and is the bilinear interpolation kernel, which can be decomposed into three one-dimensional kernels, as follows:

(8) $$B(K,K^{\prime })=b(K_{x},{K^{\prime }}_{x})\cdot b(K_{y},{K^{\prime }}_{y})\cdot b(K_{z},{K^{\prime }}_{z})$$

As shown in Figure 5, due to the three-dimensional (*x*-axis, *y*-axis, *z*-axis) transformations, the dynamic snake convolution kernel in the model covers a selectable receptive field of 9 × 9 × 9 during deformation. The application of dynamic snake convolution in the proposed registration model enhances the adaptability to complex geometric structures and improves the capability to capture key features.

During training, the 3D dynamic snake convolution adapts to the geometric shape of tubular structures by introducing deformable offsets. These offsets are updated through an iterative strategy to ensure the convolutional receptive field is focused on the target region, particularly preventing excessive shifts in the receptive field when handling elongated structures. Specifically, the offsets are calculated based on the previous position’s offset, progressively selecting the next position to maintain the continuity of the convolution kernel shape and adapt to the local features of the target. Since the offsets are typically small fractional values, trilinear interpolation is used to precisely locate each position, ensuring that the deformation of the convolution kernel aligns with the subtle snake-like shape of the structure. In this manner, the 3D dynamic snake convolution continuously adjusts the offsets during training, enhancing the model’s ability to perceive elongated and curved local features

### 3.3. The Optimization Stage

The design of the optimization phase was inspired by on-the-fly guidance [16]. In this stage, the proposed optimizer is used to iteratively refine the deformation field *ϕ* generated at the current training step. The optimized deformation field *ϕ_opt_* is then employed as a pseudo-ground truth to guide the updating of the current prediction, thereby establishing a feedback loop between the prediction model and the optimizer module. This optimization method places high demands on the deformation field output from the registration phase, as the quality of the deformation field produced by the registration model directly determines the effectiveness of the subsequent optimization process. As mentioned earlier, the registration model enhances the accuracy of the output deformation field through methods such as dynamic snake convolution, which further improves the optimization quality. The core of the optimization phase lies in minimizing the difference between the deformed image and the fixed image, which then feeds back into the 3D DSC-STUNet+ registration model to optimize the deformation field generated by the registration model.

The specific method for the optimization phase is illustrated in Figure 2. The deformation field generated during the registration phase is used as the sole parameter input into the optimization model. Through the spatial transformation network, a warped image is generated, and the loss is computed between the warped image and the fixed image. The loss function consists of two components: the local normalized cross-correlation (L_NCC_) measures the similarity between the warped image and the fixed image, while the regularization loss constrains the smoothness of the deformation field to prevent excessive distortion. An optimization algorithm (Adam) [26] is employed to minimize the loss function (L_NCC_ + L_Reg_). Using backpropagation through gradient descent, the parameters of the initial deformation field ϕ are optimized, resulting in a new optimized deformation field *ϕ_opt_*. The optimizer iterates multiple times to gradually refine the deformation field. In each training cycle, the model undergoes *n (n* = 10) iterations of optimization, with each iteration generating an optimized deformation field *ϕ_opt_* as a pseudo-ground truth deformation field, thereby implementing a weakly supervised approach. This iterative optimization process further reduces the registration error and improves accuracy. After generating the optimized deformation field, the model and deformation field are further optimized using the structural similarity index (SSIM) and regularization loss L_reg_. The SSIM is employed to enhance and preserve the model’s performance in handling local structures and image details during registration. While it increases the training time, it further strengthens the fine details processed by the registration model through 3D dynamic snake convolution.

The regularization loss L_reg_ is used to constrain the smoothness of the deformation field, preventing large deformations.

The regularization loss is implemented as follows:

(9) $$L_{reg}(\phi )=\sum_{p\in \Omega }{\left\|\nabla \phi (p)\right\|}^{2}$$

The regularization loss imposes an L2 penalty on the gradient of the deformation field to enforce its smoothness, thereby preventing excessive local distortions or unnatural deformations. Specifically, the regularization term constrains the variation in the deformation field, encouraging its smoothness and reducing unreasonable distortions. This approach enhances the quality of the registration results, particularly in terms of avoiding non-diffeomorphic deformations.

The energy function in the optimization phase of the model is composed of two terms: the image similarity loss, which measures the difference between the deformed image *I_m_* and the fixed image *I_f_*, and the regularization loss, which enforces smoothness on the deformation field *ϕ*:

(10) $$E_{opt}(I_{m},I_{f},\phi )=LNCC(I_{f},I_{m}\circ \phi )+L_{reg}(\phi )$$

where o represents the transformation function that distorts *I_m_* using *ϕ*, and the regularization term we use is the same as that in Equation (9). The similarity measure we employ in Equation (10) is the local normalized cross-correlation (NCC) between the distorted image *I_m_* and the fixed image *I_f_*:

(11) $$\begin{array}{l} LNCC(I_{f},I_{m}\circ \phi )=\\ \sum_{p\in \Omega }\frac{{\left(\sum_{p_{i}}\left(f(p_{i})-\hat{f}(p))(\left[I_{m}\circ \phi \right](p_{i})-\left[{\hat{I}}_{m}\circ \phi \right](p)\right)\right)}^{2}}{{\left(\sum_{p_{i}}\left(f(p_{i})-\hat{f}(p\right)\right)}^{2})(\sum_{p_{i}}\left[I_{m}\circ \phi \right](p_{i})-\left[{\hat{f}}_{m}\circ \phi \right](p){)}^{2})} \end{array}$$

where ${\hat{I}}_{f}$ (p) and ${\hat{I}}_{m}$ (p) represent the average voxel value within a local window of size n^3^ centered at voxel p.

The loss function of the entire optimization model is as follows:

(12) $$L_{all}=\frac{1}{n}{\sum \left(\phi -\phi_{opt}\right)}^{2}+\lambda L_{reg}$$

where *λ* is empirically set to 0.02 to ensure an optimal balance between the fidelity of the deformation fields and the regularization constraint.

**Figure 6:**
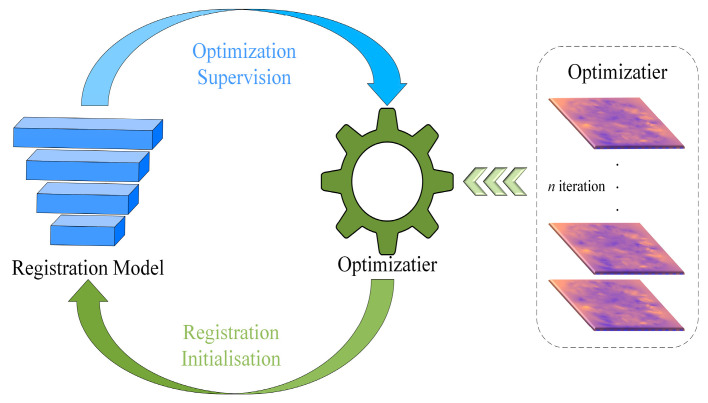
The relationship between the registration model and the optimization model.

This dynamic supervision method continuously updates with the increasing number of training epochs, enhancing the accuracy of the pseudo-ground truth through ongoing optimization during training, which provides more direct guidance for optimizing the model parameters. Furthermore, this approach exhibits self-improvement during the optimization process. As shown in Figure 6, the well-estimated deformation field obtained from the registration phase offers an advantageous initialization for the optimizer, leading to better-optimized deformation fields. This improved deformation field, in turn, provides stronger supervision for the model, establishing a positive feedback loop between prediction and optimization. As the feedback loop strengthens, the model becomes increasingly robust to large deformations and subtle anatomical variations. Ultimately, this iterative refinement process significantly improves both the stability and accuracy of the registration results.

## 4. Experiments

### 4.1. The Dataset and Experiment Setting

The datasets used in the experiment included the publicly available Information eXtraction from Images (IXI, IXI Brain Development Dataset) [27], the OASIS brain MRI dataset [28], and the LPBA (LONI Probabilistic Brain Atlas) brain MRI dataset [29]. The IXI dataset contains 576 T1-weighted brain MRI images, from which we selected 250 images for the training set, 50 for the validation set, and 100 for the test set. These images served as the fixed images in the registration task, while the moving images were taken from a brain MRI atlas, as referenced in [30]. The OASIS dataset contains 451 brain MRI images, and we selected 300 for training, 40 for validation, and 50 for testing. The training, testing, and validation images in the three datasets were randomly selected.

The IXI dataset includes various MRI modalities (T1, T2, PD, MRA, DTI) from three MRI scanners (GE, Chicago, Illinois, USA; Philips, Amsterdam, North Holland, Netherlands; Siemens, Erlangen, Bavaria, Germany), with resolutions between 1 mm^3^ and 2 mm^3^. The OASIS dataset, acquired using Siemens 1.5 T and 3 T scanners, contains T1, T2, FLAIR, DTI, and rs-fMRI images. OASIS-1 and OASIS-2 mainly have T1 MP-RAGE scans (~1 mm^3^), while OASIS-3 includes DTI (~2 mm^3^). The LPBA40 dataset, obtained with a Siemens 1.5 T scanner, has T1-weighted SPGR images (0.86 × 0.86 × 1.5 mm^3^) standardized to MNI space. For preprocessing, we used FreeSurfer v7.4.1 on the IXI dataset, applying recon-all for motion correction, skull stripping, affine registration, and intensity normalization. The brain mask was then aligned to the standard space using Talairach transformation. After segmentation of the subcortical structures, the label maps and anatomical structures were converted to .nii.gz format and transformed to ensure proper alignment with the standard space. Additionally, the dataset provides label maps of 35 anatomical structures, which were used for model evaluation. For the LPBA40 dataset, we used 30 volumes for training, 9 volumes for validation, and 1 volume as the atlas.

The experimental environment included PyTorch 2.0.0, Python 3.8 (Ubuntu 20.04), and Cuda version 11.8. The hardware setup consisted of an Intel processor with 10 vCPUs (Intel Xeon Processor) and NVIDIA A100-PCIE-40GB GPUs*2. The batch size was set to 1, the model was trained for 300 epochs using the Adam optimizer to minimize the loss function, and the global learning rate was adjusted to 1 × 10⁻⁴ when using the Adam optimizer in the registration stage. In the optimization phase, the learning rate of the Adam optimizer was set to 0.1, with a default beta value of 0.999. This configuration helped to better stabilize the variance estimation of the gradients, aiding in the refinement of the deformation field during the optimization process and improving the registration accuracy and reliability.

The choice of the number of optimization iterations *n* was explored in the subsequent ablation experiments, and the final value of *n* was determined to be 10. All images were resized to 160 × 192 × 224, and random rotations and flips were applied for data augmentation.

The performance of the registration was evaluated using the Dice coefficient as the evaluation metric. The Dice coefficient is defined as the overlap ratio between the segmentation results of the registered image and the fixed image:

(13) $$Dice(R,F)=2\cdot \frac{\left|R\cap F\right|}{\left|R\right|+\left|F\right|}$$

where *R* and *F* represent the reference image and the floating image, respectively. The Dice coefficient accurately measures the degree of overlap between two regions, thereby reflecting the quality of the registration. A perfectly overlapping image would have a Dice value of 1. Additionally, we used the Jacobian determinant to quantify changes in the topological structure and assess the smoothness of the deformation field. In the experimental results, a Jacobian determinant *J* < 0 indicates regions where the deformation deviated from being diffeomorphic.

### 4.2. Experimental Results

To validate the effectiveness and robustness of the proposed model, comparisons were made with classical registration methods, including CycleMorph [30], ViT-V-Net [11], VoxelMorph [9], TransMorph [12], NiftyReg [31], and Syn [32]. The Dice coefficient was employed to quantify the overlap between the moving image and the fixed image after registration, while the Jacobian determinant was used to measure changes in topological structure and assess the smoothness of the deformation field.

**Figure 7:**
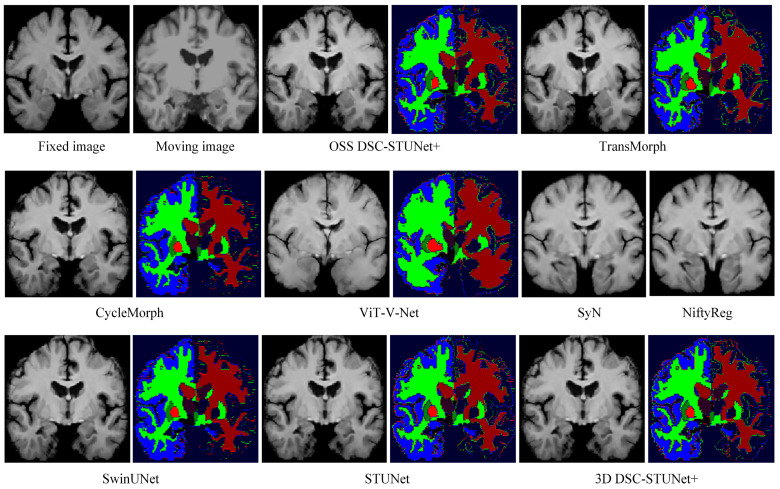
Registration results and deformation fields of different methods on the IXI dataset (z = 124).

**Figure 8:**
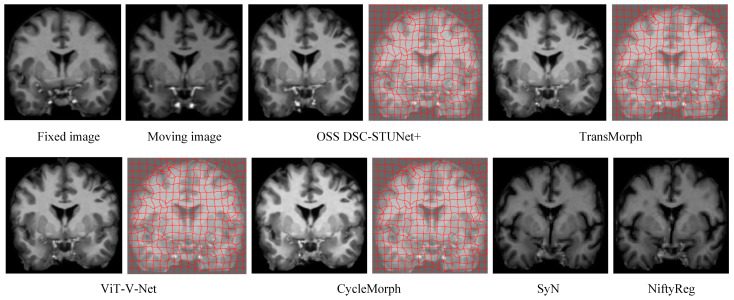
Registration results and segmented images of different methods on the OASIS dataset (z = 124).

From Table 1, it can be observed that the proposed model achieved a Dice score on the IXI dataset that was 7.0% higher than CycleMorph, 6.6% higher than VoxelMorph, 16.3% higher than NiftyReg, 16.2% higher than SyN, 4.8% higher than ViT-V-Net, and 2.3% higher than TransMorph. From Table 2, the model’s Dice score on the OASIS dataset was 5.3% higher than CycleMorph, 4.8% higher than VoxelMorph, 9% higher than NiftyReg, 7.5% higher than SyN, 4.2% higher than ViT-V-Net, and 1.8% higher than TransMorph. From Table 3, on the LPBA40 dataset, the proposed model’s Dice score was 7% higher than CycleMorph, 5.9% higher than VoxelMorph, 1.1% higher than NiftyReg, 1.7% higher than SyN, 5.4% higher than ViT-V-Net, and 2.6% higher than TransMorph. Figure 7 and Figure 8 present the registration results of the different methods on the IXI dataset and OASIS dataset, respectively. From Figure 7 and Figure 8, it can be observed that the proposed method not only exceled in global accuracy compared to the conventional registration methods but also better preserved the details in the images.

In Figure 7, the feature segmentation map for the IXI dataset shows that the model demonstrated a higher level of attention to complex and irregular edges than models like TransMorph. The advantage of OSS DSC-STUNet+ over the other methods lay in its ability to extract both global and local features, which can be validated through the registration images and their corresponding feature segmentation maps.

### 4.3. Ablation Experiment

In order to verify the improvement in the optimization model over the training capability of the registration model and the quality of image registration, as well as to assess the enhancement in the extraction of local fine features using the 3D dynamic snake convolution and the ability of the multi-scale dense skip connection to improve the global feature extraction of the SwinUNet model, ablation experiments were conducted on the IXI, OASIS, and LPBA40 datasets. The ablation experiments used SwinUNet as the baseline network. In the OSS DSC-STUNet+ network model, the DSConv3D in the DSC-STUNet part of the registration module was replaced with Conv3D, named STUNet. Additionally, the dense skip connections in 3D DSC-STUNet+ were replaced with ordinary skip connections, named 3D DSC-STUNet. The Dice values from the ablation experiments are shown in Table 4, Table 5 and Table 6, and the result images on the IXI dataset are displayed in Figure 7.

The tables list the Dice values for the five models. The experimental results indicate that the methods proposed in this paper all improved the performance of the registration network. By increasing the depth of the SwinUNet network and the number of skip connections, the STUNet model was able to achieve a 1.5–1.7% improvement in Dice score. Replacing Conv3D with DSConv3D further improved the Dice score by 0.3–0.4%, validating the applicability of the DSConv3D module. Additionally, Figure 8 shows that the registration results of the 3D DSC-STUNet were more accurate than those of STUNet and SwinUNet, particularly in the handling of the fine white matter edges in the segmented images.

**Figure 9:**
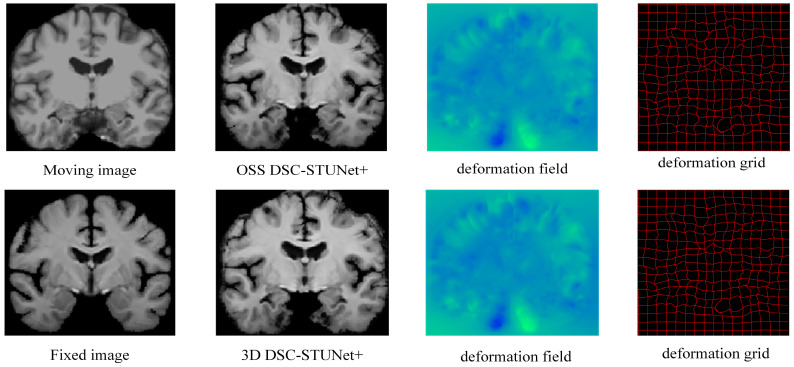
The registration results, deformation field, and deformation grid of the registration model before and after optimization.

The introduction of weakly supervised learning and optimization methods into the registration model resulted in a 0.7–4.1% increase in Dice score and reduced the percentage of non-diffeomorphic voxels (|*J_φ_*| < 0) by half. This demonstrates that OSS not only improved the model performance but also prevented the generation of overly sharpened deformation fields, which can be visually observed by comparing the actual registration results in Figure 7. In the Figure 9, we illustrates the registration results, deformation fields, and deformation grids of the OSS DSC-STUNet+ model with the application of the weakly supervised optimization strategy, compared to the 3D DSC-STUNet+ model without the weakly supervised strategy. The figure shows that the optimized deformation field captured finer details, while preserving good local details, and the global registration performance was also partially improved.

In conclusion, the analysis of the experimental results in Figure 7 confirms that introducing 3D dynamic snake convolution, utilizing multi-scale dense skip connections, increasing the model’s depth and the number of skip connections, and employing weakly supervised strategies and optimization methods during the training of deep learning models can lead to higher Dice scores in MRI image registration. The registration results were more accurate, and the model’s handling of image details was more precise, ultimately leading to enhanced model performance. These improvements demonstrate the potential of the proposed approach for tackling complex medical image registration tasks.

To validate the improvement in registration performance of the 3D dynamic snake convolution (DSConv3D) compared to other types of convolutions, the DSConv3D in the 3D DSC-STUNet+ was replaced with large kernel convolution (LKC) [33], dilated convolution (DC) [34], sparse convolution (SC) [35], and standard 3D convolution (Conv3D), respectively. The experimental results are shown in Figure 9.

**Figure 10:**
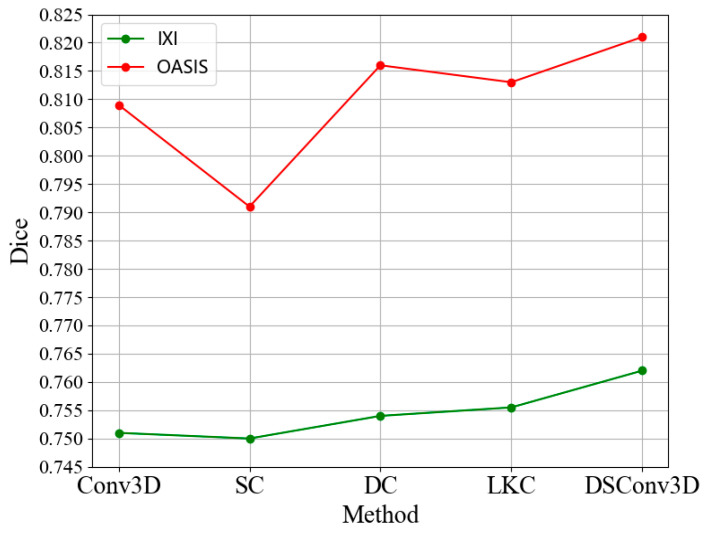
Dice scores of the different convolutions tested in the model.

As seen in Figure 10, for the IXI dataset, sparse convolution (SC) yielded the lowest Dice score among the five types of convolution. The Dice scores for dilated convolution (DC) and large kernel convolution (LKC) were 0.2% and 0.3% higher, respectively, than the results using standard convolution. The 3D dynamic snake convolution (DSConv3D) achieved the best Dice score, with similar results observed for the OASIS dataset. These findings demonstrate that 3D dynamic snake convolution provided superior performance in the proposed registration model, as replacing standard convolution with dynamic snake convolution increased the Dice score by 1.46%.

## 5. Discussion

This study aimed to evaluate the performance of the proposed model on various MRI datasets. The key focus of medical image registration evaluation lies in assessing whether the generated deformation field can effectively align the fixed and moving images, ensuring alignment accuracy between anatomical regions (such as organs or tissues) after registration. The Dice similarity coefficient, commonly used in segmentation registration, served as the primary evaluation metric. In experiments on the IXI dataset, the proposed model achieved a Dice score improvement ranging from 2.3% to 16.4% compared to other classical registration models. On the OASIS dataset, the improvement ranged from 1.8% to 9%, while on the LPBA40 dataset, the improvement was between 2.6% and 7%. These experimental results demonstrate that the proposed model outperformed the other classical registration models in terms of Dice score. Moreover, by comparing the experimental results in Figure 7 and Figure 8, it is evident that the proposed model exhibited stronger detail handling capabilities compared to other classical models.

In addition, as shown in Table 7, we also measured the inference times of several classical methods, along with the pre-optimization 3D DSC-STUNet+ and the optimized OSS DSC-STUNet+ models on the IXI dataset. From the table, it can be observed that our method inference time remained comparable to the other methods. Furthermore, the inference efficiency of the model remained unchanged before and after optimization, demonstrating the clinical applicability of the weakly supervised optimization strategy.

For the 3D dynamic snake convolution (DSConv3D), due to its relatively high memory requirements, we introduced it at three specific positions within the model. Ablation experiments revealed that incorporating DSConv3D at these three positions resulted in a 1.1–2.1% improvement in Dice scores for the 3D DSC-STUNet model compared to the STUNet model across the IXI, OASIS, and LPBA40 datasets. Figure 7 demonstrates that the 3D DSC-STUNet model, after incorporating DSConv3D, provided more detailed and enriched results on the IXI dataset.

**Figure 11:**
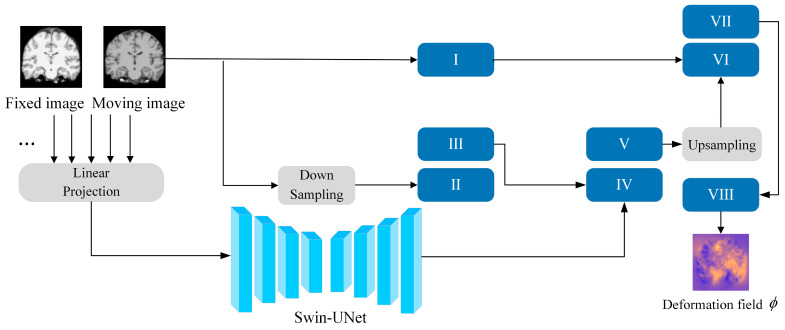
Before introducing the 3D dynamic snake convolution, the 3D DSC-STUNet+ model had a total of eight ordinary convolutions, numbered from I to VIII. All eight convolution positions can be replaced with 3D dynamic snake convolutions instead of ordinary convolutions.

**Figure 12:**
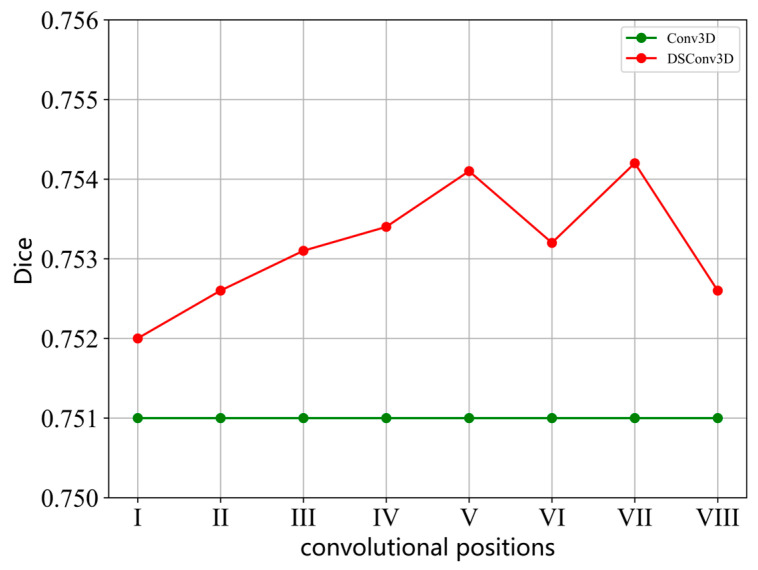
The Dice values of the dynamic snake convolution tested at different positions in the model.

We further investigated the impact of introducing DSConv3D at different positions within the model. The proposed model has eight potential positions where convolutions can be replaced with DSConv3D, labeled as I, II, III, …, VIII in Figure 11. Experiments were conducted by individually replacing the convolutions at each of these positions with DSConv3D, while keeping the standard Conv3D at the remaining positions. The Dice score results are presented as a line chart in Figure 12. From Figure 12, it is observed that incorporating DSConv3D at position I only increased the Dice score by 0.13% compared to STUNet, while its placement at positions II or VIII resulted in a 0.19% improvement. However, at positions III, IV, and VI, the Dice score improvement reached 0.32%, and at positions V and VII, the improvement reached 0.41%. When DSConv3D was simultaneously introduced at positions V and VII, further experiments were conducted to compare the effects of adding DSConv3D to positions III, IV, and VI. The results indicated that the highest Dice score was achieved when DSConv3D was incorporated at positions V, VII, and III, with a 1.5% improvement compared to STUNet. Nevertheless, the high memory demands of DSConv3D remain a limitation and require further optimization.

In the experiment, we found that after introducing dynamic snake convolution, the memory usage of the model increased by 37,286 MiB compared to before, indicating that the model has a high demand for GPU memory. With 3D dynamic snake convolution, several optimization strategies can be used to reduce the memory consumption. Grouped convolution can divide the input channels into multiple groups to ease the computational burden. Additionally, using nearest-neighbor interpolation or reducing the interpolation points can lower the memory overhead. A sparse update strategy, where offset calculations are only performed in regions with significant deformations, minimizes redundant computations. These techniques aim to maintain the computational efficiency of 3D dynamic snake convolution, while alleviating the high memory demands, with further evaluations to be conducted in upcoming experiments.

In the optimization part of the model, the ablation experiments showed that, compared to the non-optimized 3D DSC-STUNet model, the optimized model achieved a 0.7–4.1% improvement in Dice scores across the three datasets. By applying more precise registration, the deformation field predicted by the model provided additional supervision, enhancing the model’s accuracy in the registration task. In the loss function, we used a combination of SSIM and L_reg_ losses, not only to strengthen the details and structure of the optimized deformation field, but also to prepare for the future integration of multi-modal data into our model.

**Figure 13:**
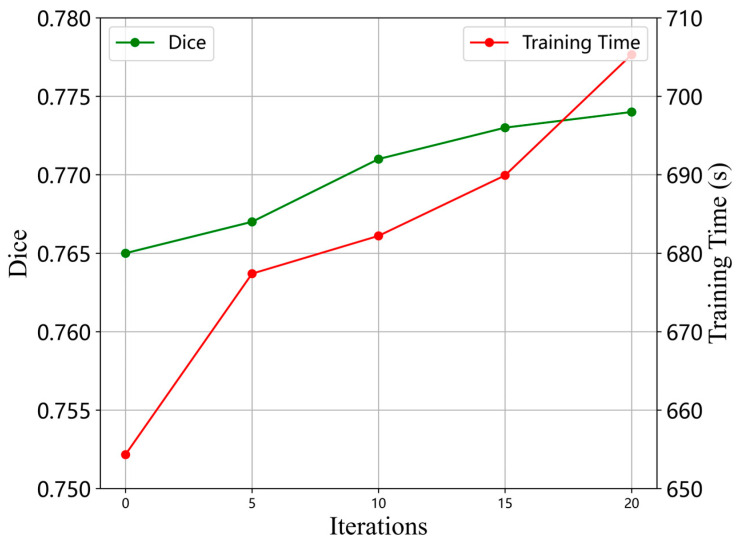
Dice scores and training time of models with different iteration settings.

Furthermore, to investigate the relationship between the number of optimizer iterations, the final Dice score, and the training time, we set the number of iterations to 1, 5, 10, 15, and 20, and computed the average training time per epoch for each iteration count. Training and validation were conducted on the IXI dataset. As shown in Figure 13, the experiment confirmed that the model’s Dice score improved as the number of iterations increased. By calculating the ratio of Dice score to training time, we found that among the five randomly selected iteration counts, the ratio was highest when the iteration count was set to 10, which corresponded to the lowest relative time cost for model training. Therefore, setting the iteration count to 10 stuck a good balance between registration performance and training time, yielding satisfactory results without significantly prolonging the training duration.

In weakly supervised medical image registration, progressive pseudo-label refinement is an effective strategy to ensure the quality and reliability of the pseudo-ground truth deformation fields. Initially, only high-confidence pseudo-deformation fields are used for training, to minimize the impact of incorrect labels. As training progresses, newly generated pseudo-deformation fields are gradually incorporated, and the previously optimized deformation fields serve as supervision signals to dynamically update the pseudo-labels. This adaptive refinement process enables the model to correct pseudo-labels iteratively, preventing erroneous convergence and enhancing the stability and accuracy of image registration.

## 6. Conclusions

In summary, this paper presented an optimization-based weakly supervised deformable medical image registration method, namely OSS DSC-STUNet+. This method was built upon the Swin-UNet model, enhancing the model’s ability to process image details through the use of multi-fusion dense skip connections in the skip links of the swin transformer component. Subsequently, further upsampling and skip connections were incorporated into the model, along with a three-dimensional dynamic snake convolution, effectively extracting both global and local features from moving and fixed images. The model employs a weakly supervised approach, utilizing the deformation fields generated during training as pseudo-ground truth labels to supervise the model, thereby ensuring registration accuracy. Experimental results on the IXI, OASIS, and LPBA40 datasets demonstrated the superiority of the proposed method in terms of visual assessment and Dice quantification metrics.

Additionally, we plan to incorporate new attention mechanisms to further improve the registration accuracy and efficiency, and we will focus on extending this method to multimodal medical image registration in future works, as well as integrating physiological structural imaging from other modalities to enhance the model’s interpretability. We aim to develop OSS DSC-STUNet+ into a more versatile and robust framework for medical image registration, providing strong support for clinical diagnosis and treatment.

## Figures and Tables

**Table 1:** Dice values of different models on the IXI dataset.

Method	% of |*J*_*ϕ*_| ≤ 0	Dice
CycleMorph	1.071 ± 0.382	0.717 ± 0.183
VoxelMorph	0.911 ± 0.079	0.720 ± 0.114
NiftyReg	0.011 ± 0.046	0.645 ± 0.163
SyN	<0.0001	0.646 ± 0.144
ViT-V-Net	0.887 ± 0.197	0.734 ± 0.121
TransMorph	0.765 ± 0.169	0.754 ± 0.124
OSS DSC-STUNet+	0.409 ± 0.028	0.771 ± 0.141

**Table 2:** Dice values of different models on the OASIS dataset.

Method	% of |*J*_*ϕ*_| ≤ 0	Dice
CycleMorph	0.469 ± 0.161	0.785 ± 0.197
VoxelMorph	0.517 ± 0.134	0.789 ± 0.107
NiftyReg	<0.0001	0.762 ± 0.152
SyN	<0.0001	0.767 ± 0.144
ViT-V-Net	0.577 ± 0.121	0.794 ± 0.121
TransMorph	0.521 ± 0.199	0.814 ± 0.164
OSS DSC-STUNet+	0.436 ± 0.047	0.829 ± 0.154

**Table 3:** Dice values of different models on the LPBA40 dataset.

Method	% of |*J*_*ϕ*_| ≤ 0	Dice
CycleMorph	0.796 ± 0.152	0.653 ± 0.191
VoxelMorph	0.691 ± 0.127	0.658 ± 0.121
NiftyReg	<0.0001	0.691 ± 0.141
SyN	<0.0001	0.687 ± 0.104
ViT-V-Net	0.657 ± 0.144	0.661 ± 0.169
TransMorph	0.621 ± 0.135	0.681 ± 0.153
OSS DSC-STUNet+	0.471 ± 0.047	0.699 ± 0.124

**Table 4:** Results of ablation experiments on IXI dataset.

Method	Dice
SwinUNet	0.737 ± 0.156
STUNet	0.751 ± 0.114
3D DSC-STUNet	0.762 ± 0.147
3D DSC-STUNet+	0.764 ± 0.103
OSS DSC-STUNet+	0.771 ± 0.141

**Table 5:** Results of ablation experiments on OASIS dataset.

Method	Dice
SwinUNet	0.795 ± 0.117
STUNet	0.809 ± 0.192
3D DSC-STUNet	0.821 ± 0.124
3D DSC-STUNet+	0.823 ± 0.146
OSS DSC-STUNet+	0.829 ± 0.154

**Table 6:** Results of ablation experiments on LPBA40 dataset.

Method	Dice
SwinUNet	0.657 ± 0.133
STUNet	0.661 ± 0.174
3D DSC-STUNet	0.669 ± 0.181
3D DSC-STUNet+	0.670 ± 0.153
OSS DSC-STUNet+	0.699 ± 0.124

**Table 7:** Inference time of the different models on the IXI dataset.

Method	T*_infer_*(s)
CycleMorph	0.512 (GPU)
VoxelMorph	0.070 (GPU)
NiftyReg	27.44 (CPU)
SyN	301.1 (CPU)
ViT-V-Net	0.619 (GPU)
TransMorph	0.121 (GPU)
3D DSC-STUNet+	0.311 (GPU)
OSS DSC-STUNet+	0.311 (GPU)

## Data Availability

The raw data supporting the conclusions of this article will be made available by the authors on request.
